# The Cost of Car Upkeep

**Published:** 1909-12-25

**Authors:** 


### Motoring Notes.

### THE COST OF CAR UPKEEP.

I have been asked to give a few particulars as to the cost of running a small car, but as this depends on so many different factors any estimate approaching exactitude is wholly impossible. Letters are constantly appearing in the motor journals from owners giving their expenses for car upkeep, sometimes for a year and sometimes for a number of years, but as these communications refer to cars of different make, size, and power they are useless as a means of estimating the cost of running a car suitable for the average medical man. The difficulty of the subject will be further realised when one considers the very strong bearings on the question of expense, of skill in driving, and the care taken of the car. A larger proportion of the expense of upkeep depends on the care and attention of the owner or driver than on the cost of petrol, tyres, etc.

The only reliable records on which to base an estimate are, I think, the returns of expenses forwarded to a well-known motor firm by doctors who use their small cars. The replies they received, being the actual experiences under the normal conditions of doctors' use, are most valuable when summarised both to the actual and prospective medical motorist. In reproducing them, I may say that they include all expenses except driver or cleaner, motor-house, and depreciation, which vary according to circumstances.

*Petrol.*—The reports show a total mileage of 97,379, and the cost of petrol £263 3s. 6d. Averaging the price paid for petrol at 14d. a gallon, it means a consumption of 4,511 gallons at the rate of 21.617 miles per gallon.

*Lubricating Oil and Grease.*—In the reports where the expenses for oil and grease are quoted the mileage amounts to 93,299 and the cost £48 4s. 2d.—0.12d. a mile.

*Tyre Repairs and Replacements.*—In the reports where these items are stated the mileage totals 92,879, and the costs £210 15s. 6d.—0.54d. a mile.

*Mechanical Repairs and Replacements.*—In the reports where these items are separated from others the mileage totals 97,379, and the cost £124 16s. 3d. —0.3d. a mile.

Based on the average of the above analysis, the cost of running a small doctor's car 5,000 miles, which is about the distance travelled in one year by a doctor, would be:—

| | £ | s. | d. |
|---|---|---|---|
| Petrol ... ... ... ... ... ... | £13 | 10 | 2 |
| Lubricating oil and grease ... ... | 2 | 11 | 8 |
| Mechanical repairs, etc. ... ... ... | 6 | 4 | 2 |
| Tyre repairs and replacements ... ... | 11 | 6 | 11 |
| Sundries ... ... ... ... ... | 1 | 9 | 1 |
| Carriage and driving licenses ... ... | 2 | 7 | 0 |
| Insurance ... ... ... ... ... | 8 | 5 | 0 |
| | £45 | 14 | 0 |

This sum may be safely taken as a guide as to the expense of running a small car for a year. Possibly, however, the expenses during the first year should prove considerably less, since if tyres of a large section were fitted the cost under this heading should be reduced to a few shillings for repairs of punctures. There are now many garage proprietors who are prepared, when they sell a car of this type, to find petrol, oil, tyres, keep the car in good running order, and insure same against all risks for the sum of £35 the first year, £45 the second and third years, and £55 the fourth and fifth years. Where the trade is prepared to do this, the cost of keeping a small car can be accurately estimated. It is a system which is now largely growing in favour with medical men and it will be recognised that there is much to recommend it to members of the profession.

